# PCR based bronchoscopic detection of common respiratory pathogens in chronic cough: a case control study

**DOI:** 10.1186/1745-9974-8-5

**Published:** 2012-09-14

**Authors:** Peter W West, Angela Kelsall, Samantha Decalmer, Winifred Dove, Paul W Bishop, James P Stewart, Ashley A Woodcock, Jaclyn A Smith

**Affiliations:** 1 Respiratory Research Group, Faculty of Medical and Human Sciences, The University of Manchester, Manchester Academic Health Science Centre, Manchester, UK; 2 NIHR Translational Research Facility in Respiratory Medicine, North West Lung Research Centre, University Hospital of South Manchester, Manchester, UK; 3 Department of Clinical Infection, Microbiology and Immunology, Duncan Building, The University of Liverpool, Liverpool, UK; 4 Directorate of Clinical Laboratory Medicine, University Hospital of South Manchester, Manchester, UK; 5 Department of Infection Biology, Duncan Building, The University of Liverpool, Liverpool, UK; 6 Respiratory Research Group, 2nd Floor Education and Research Centre, University Hospital of South Manchester, Southmoor Road, Manchester, M23 9LT, UK

**Keywords:** Biopsy, Bronchoalveolar lavage, Bronchoscopy, Cough, Infection, PCR, Virus

## Abstract

**Background:**

Viral respiratory tract infection is the most frequent cause of acute cough and is reported at onset in about one third of patients with chronic cough. Persistent infection is therefore one possible explanation for the cough reflex hypersensitivity and pulmonary inflammation reported in chronic cough patients.

**Methods:**

Bronchoscopic endobronchial biopsies and bronchoalveolar lavage cell counts were obtained from ten healthy volunteers and twenty treatment resistant chronic cough patients (10 selected for lavage lymphocytosis). A screen for known respiratory pathogens was performed on biopsy tissue. Chronic cough patients also underwent cough reflex sensitivity testing using citric acid.

**Results:**

There was no significant difference in incidence of infection between healthy volunteers and chronic cough patients (p = 0.115) or non-lymphocytic and lymphocytic groups (p = 0.404). BAL cell percentages were not significantly different between healthy volunteers and chronic cough patients without lymphocytosis. Lymphocytic patients however had a significantly raised percentage of lymphocytes (p < 0.01), neutrophils (p < 0.05), eosinophils (p < 0.05) and decreased macrophages (p < 0.001) verses healthy volunteers. There was no significant difference in the cough reflex sensitivity between non-lymphocytic and lymphocytic patients (p = 0.536).

**Conclusions:**

This study indicates latent infection in the lung is unlikely to play an important role in chronic cough, but a role for undetected or undetectable pathogens in either the lung or a distal site could not be ruled out.

**Trials registration:**

Current Controlled Trials ISRCTN62337037 & ISRCTN40147207

## Background

Cough is a common reason for patients to seek medical attention [1]. It is well known that viral infection of the upper respiratory tract is the most common cause of acute cough [2] and an increase in cough reflex sensitivity has been demonstrated during upper respiratory tract infections (URTI) in healthy subjects, similar to that seen in patients with chronic cough [3,4]. Most respiratory infections which lead to cough are caused by viruses such as picornaviruses, coronaviruses, parainfluenza, influenza A and B, human metapneumovirus, respiratory syncytial viruses and adenoviruses. But they may also be caused by other pathogens such as human bocavirus or mycoplasma [5-8].

About one third of chronic cough patients recall an URTI at the onset of their cough, in one series 34% [9] and in our clinic approximately 30% (unpublished data). This suggests, that whilst the vast majority of URTI are self-limiting, some may persist to cause a troublesome chronic cough [10]. Although concomitant conditions that could explain coughing (i.e. nasal disease, reflux disease, asthma) can be identified in a proportion of chronic cough patients, many of them respond poorly to specific therapy [11]. Furthermore the majority of patients presenting with typical symptoms of these conditions do not complain of severe cough, suggesting that whilst participating in triggering cough events, they may not be relevant to the development of chronic cough. Other, largely unknown, factors may be driving cough hypersensitivity and the associated inflammation.

The mechanisms by which respiratory tract infections might affect cough reflex sensitivity are not clear. Certainly, the neuroinflammatory paradigm of inflammatory actions on peripheral nerve sensitization is well established in acute models of inflammatory pain and chronic bowel diseases. As similar unmyelinated C-fibre and thinly myelinated Aδ-fibre afferent nerves innervate the airways, skin and viscera it is tempting to infer a similar aetiology in cough. Cytokines, neuropeptides, growth factors and eicosanoids might all be important mediators.

In chronic cough, studies have established varying degrees of airway remodelling and inflammation. Some patients present with BAL lymphocytosis with increased numbers of activated CD4^+^ Th1 cells [12], a phenomenon associated with autoimmune disease in chronic cough [13]. Infiltration of lymphocytes as a result of viral infection can sensitize neural pain responses in post-herpetic neuralgia [14] and respiratory infections are linked to neurological syndromes such as Guillain–Barré [15,16]. Additionally, infection in murine models increases neuropeptide release which could activate neurokinin-1 receptors (NK1R) on both CD4^+^ T-cells [17], and sensory nerve terminals, reducing the excitation threshold for action potential initiation [18]. Both mycoplasmal and viral infections have been linked with the development of autoimmune reactions which could lead to CD4^+^ autoreactive T-cell mediated mucosal damage [19,20]. The presence of infection could also stimulate the production of cytokines such as TGF-β [21] and CXCL8 from macrophages and epithelial cells as well as activate mast cells.

We therefore hypothesized that sub-clinical chronic airway infections might contribute to heightened cough reflex sensitivity and inflammation in patients with chronic cough [21-23]. Moreover we predicted that increased bronchoalveolar lavage (BAL) lymphocytes might be indicative of such infections. We conducted a PCR based screen for known pathogens in biopsy tissue of 10 healthy volunteers and 20 treatment resistant chronic cough patients, of which 10 were selected for BAL lymphocytosis (greater than 20%).

## Methods

### Subjects

Patients were recruited from those referred to a specialist cough clinic (University Hospital of South Manchester, UK) complaining of chronic cough (>8 week duration) between 2005 and 2007. Current smokers and ex-smokers of <6 months, patients with significant co-morbidities (e.g. chronic obstructive pulmonary disease, diabetes, heart disease), and those receiving angiotensin converting enzyme inhibitors or opiates were excluded. Final diagnoses were based on investigation findings (pulmonary function tests and cough assessments) and treatment trials were given in accordance with the British Thoracic Society guidelines [24]. Healthy volunteers were recruited through response to local advertising.

As this is the first study to our knowledge, to address the potential role for infection in chronic cough, it was impossible to conduct a power calculation prior to commencing. Therefore the numbers of subjects used was selected on the basis of similar published studies in other disease areas [25].

### Study design and procedures

The study samples consisted of BAL and endobronchial biopsies from 10 healthy volunteers and 20 chronic cough patients (10 retrospectively selected for BAL lymphocytosis >20%) with informed consent according to protocols approved by North and South Manchester Research Ethics Committees (LREC References 07/Q1406/15 and 05/Q1403/117). The study samples from chronic cough patients were chosen at random, after stratification based on the BAL lymphocyte counts, from those obtained from a cohort of 100 patients, all of whom had undergone bronchoscopy as part of their clinical investigations for chronic cough. Only data pertaining to those 20 patients is presented in this manuscript.

#### Bronchoscopy

Bronchoscopy was performed under conscious sedation with topical lidocaine applied to the nose, larynx and airways. BAL was collected from the right middle lobe (3 aliquots, 60 ml normal saline). Endobronchial biopsies were from the carinae of the basal segments of the right lower lobe.

#### BAL cell counts

BAL cell counts were performed according to standard operating procedures by the clinical cytology laboratory at the University Hospital of South Manchester. The cell pellet from a 10 ml aliquot of BAL was washed, counted and resuspended at a concentration of 0.02 x 10^6^ cells.ml^-1^. Haematoxylin and eosin stained cytospins were prepared. A differential cell count was performed by counting 300 cells and results expressed as a percentage.

#### Processing of biopsies for viral nucleic acids

Biopsies were snap frozen at the point of sampling and stored at −80°C. Nucleic acids were extracted from whole tissue using RNeasy and QIAamp extraction kits (Qiagen, Crawley, UK). Nucleic acid quality was assessed using a 2100 bioanalyzer (Agilent technologies) and only samples containing high quality DNA and RNA were used. For RNA, an RNA integrity number (RIN) of greater than five was required. PCR based assays for DNA or RNA from infectious organisms were carried out according to methods we have used and published previously [7,26]. Further details can be found in an additional file [see Additional file 1.

#### Cough reflex sensitivity testing

A citric acid cough challenge test was performed using an ascending dose (0.03 – 4 M) dosimeter method as previously described [27]. Six 12 μl single breath inhalations of citric acid, with three additional randomly interspersed placebo (normal saline) doses, were delivered. The number of coughs was counted by an experienced observer for one minute after each inhalation. The challenge test stopped after the concentration of citric acid eliciting at least 5 consecutive coughs (C5) was reached.

### Statistical analysis

Analyses were performed using Prism (Version 5, Graphpad, San Diego, CA) or SPSS (Version 15, IBM, Armonk, New York). Data are expressed as Mean (+/− S.E.M) or Median (I.Q.R. 25th-75th percentile). Categorical data were compared using Fisher’s exact test. Cough reflex sensitivity data were logarithmically transformed and compared using an independent samples t-test. Non-parametric data were analysed using the Mann–Whitney U-test. Multiple comparisons were made using One-way ANOVA and Tukey’s post-test correction or Kruskal-Wallace test with Dunn’s post-test correction as appropriate. Significant differences are presented as * = p < 0.05, ** = p < 0.01, *** = p < 0.001.

## Results

### Subjects

The characteristics of the study participants are shown in Table 1. The healthy volunteers were younger than the chronic cough patients (p < 0.001) and fewer were ex-smokers, although this was not significant (p = 0.372). Other group demographics were comparable. The concomitant conditions, potentially contributing to cough are shown in Table 2. Patients were treated, simultaneously for all concomitant conditions that may be triggering cough, as per BTS guidelines [24]. Of the 20 patients, 10 rated their response to this therapy as good, 5 reported a partial response, 4 no response and 1 subject was lost to follow up. No pathogen was detected in any of the non-responders. 

**Table 1:** Study Subject Characteristics

	**Healthy volunteers**	**Chronic cough**			**HV vs. all patients (P-value)**
		**All patients**	**Lymphocytes <10%**	**Lymphocytes >20%**	
*n*	10	20	10	10	-
Age (yr)	31.0 (29.25-42)	57.0 (51–61.75)	57.5 (50.5-59.75)	57.0 (51.5-64.75)	*P < 0.001*^†^
Male, *n*	5	7	4	3	P = 0.461^◊^
Cough Duration (yr)	n/a	6.6 (1.64)	5.9 (1.99)	7.3 (2.69)	-
Smoking status					
* Never*	9	14	7	7	P = 0.372^◊^
* Ex*	1	6	3	3	
* Pack Years*	1.0	7.0 (3–9.5)	8.0 (7–9)	2.0 (1.5-12.5)	P = 0.356^†^
BAL lymphocyte %	9.5 (6.25-12.5)	15.5 (6.5-30.25)	6 (3.5-8.5)	30.5 (22–32)	P = 0.402^†^

Data expressed as mean (SEM) or Median (IQR, 25th-75th Percentile). Data were compared using Mann–Whitney U-test (†) or Fisher’s exact test (◊).

**Table 2:** Concomitant conditions potentially contributing to cough identified in study subjects

		**Chronic cough**		
	**Healthy volunteers**	**All patients**	**Lymphocytes <10%**	**Lymphocytes >20%**
*n*	10	20	10	10
Concomitant Conditions				
* PND*	-	6	3	3
* PND & Asthma*	-	1	-	1
* Reflux Disease*	-	3	1	2
* Reflux & EB*	-	1	-	1
* EB*	-	1	1	-
* TPO*	-	1	1	-
* No Trigger*	-	5	3	2
* Bronchiectasis*	-	1	-	1
* Spontaneous resolution*	-	1	1	-

PND = post nasal drip, EB = eosinophilic bronchitis, TPO = tracheopathia *osteochondroplasia.*

### BAL cell counts

**Figure 1:**
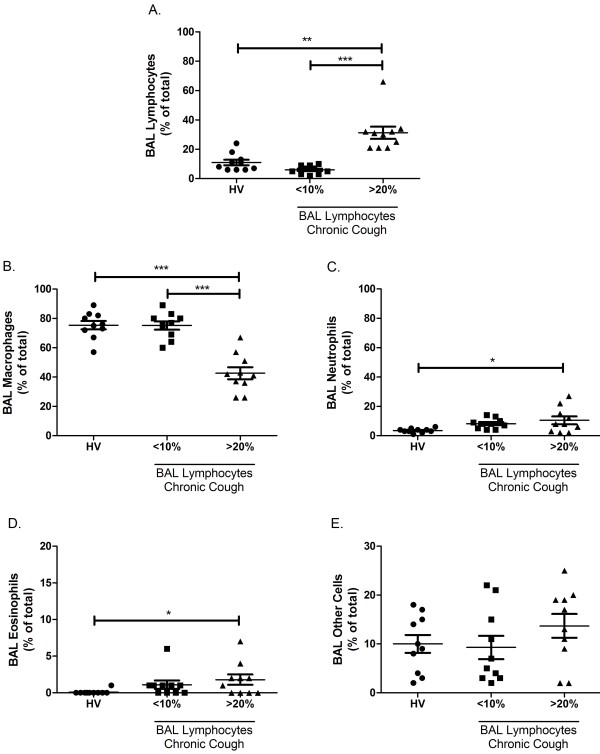
**BAL Cell Percentages. **Percentage of lymphocytes (**A**), macrophages (**B**), neutrophils (**C**), eosinophils (**D**) and other cell types (**E**) in BAL. Differential cell counts were performed by the clinical cytology laboratory on haematoxylin & eosin stained cytospins. A total of 300 cells were counted on each slide. Data are presented as % total cell count for each individual data point. Error bars indicate mean +/− S.E.M. or Median with I.Q.R. * = p < 0.05, ** = p < 0.01, *** = p < 0.001.

BAL cell percentages are shown in Figure 1. As expected, the BAL lymphocyte percentage in the patients with lymphocytosis >20% were highly significantly different from both healthy volunteers and chronic cough patients without lymphocytosis (Figure 1, A). The percentage BAL lymphocytes in healthy volunteers and non-lymphocytic patients were not significantly different. The percentage of BAL macrophages were significantly reduced in the lymphocytic population. This is likely a consequence of the raised lymphocyte percentage (Figure 1, B). BAL neutrophils were slightly raised in the lymphocytic cough patients when compared to healthy volunteers (Figure 1, C). There is a similar trend towards slightly raised eosinophils in the chronic cough groups, although this was only significant for the lymphocytic population (Figure 1, D). There was no significant difference in BAL lymphocytes between individuals with or without detected pathogens, within the sample as a whole, or within the chronic cough patients (p = 0.928 and 0.368 respectively, Mann–Whitney U-test).

### Endobronchial biopsies

Results of the screen for nucleic acids from infectious organisms are shown in Table 3. There was no significant difference between the incidence of positive PCR for infectious agents between healthy volunteer and chronic cough groups (p = 0.115), or between the healthy, non-lymphocytic and lymphocytic groups (p = 0.404). Two samples from patients with BAL lymphocytosis had a low level positive result for Epstein-Barr virus. These were the only documented pathogens in this sample group. Whilst healthy and non-lymphocytosis controls were negative for Epstein-Barr, two samples from the patient control group and three from the healthy volunteers tested weakly positive for *Chlamydophila sp*. In addition, one healthy volunteer sample tested positive for influenza A and another for adenovirus. No study subjects tested positive for co-incident infection or developed a clinical infection following bronchoscopy.

**Table 3:** Samples where nucleic acids (RNA or DNA) to infectious organisms were identified

		**Chronic cough**			
	**Healthy volunteers**	**All patients**	**Lymphocytes <10%**	**Lymphocytes >20%**	**P**
RSV	-	-	-	-	ns
hMPV	-	-	-	-	ns
Influenza A & B	1 positive	-	-	-	ns
PIV	-	-	-	-	ns
HCoV	-	-	-	-	ns
Rhinovirus	-	-	-	-	ns
EBV	-	2 positive	-	2 positive	ns
HBoV	-	-	-	-	ns
Adenovirus	1 positive	-	-	-	ns
*Chlamydophila sp.*	3 positive	2 positive	2 positive	-	ns
*Mycoplasma pn.*	-	-	-	-	ns
VZV	-	-	-	-	ns

There was no overall significant difference between healthy volunteer and chronic cough groups (Fisher’s exact test). Ns = not significant (p > 0.05, Fisher’s exact test). RSV = respiratory syncytial virus, hMPV = human metapneumovirus, PIV = parainfluenza virus, HCoV = human coronavirus, EBV = Epstein-barr virus, HBoV = human bocavirus, VZV = varicella zoster virus.

A retrospective power calculation suggested that with this sample size the study would have 80% power to detect a prevalence of 50-55% for any individual pathogen in the chronic cough patient group, assuming a prevalence of <1% in the healthy controls.

### Cough reflex sensitivity

**Figure 2:**
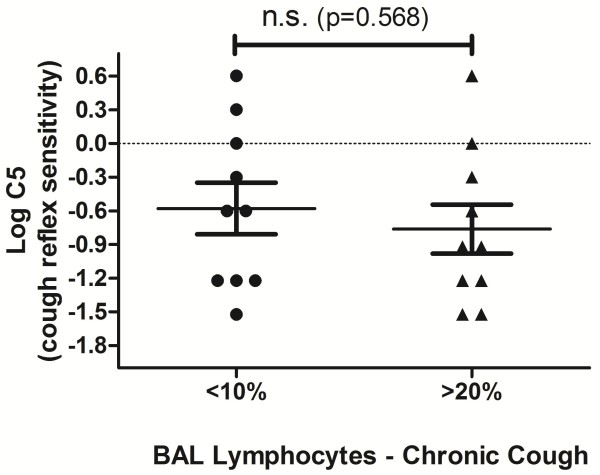
**Cough Reflex Sensitivity Test. **Cough reflex sensitivity (log_10_ [C5]) to citric acid in chronic cough patients was performed using an ascending dose (0.03 – 4 M) with three additional randomly interspersed placebo (normal saline) controls. The number of coughs was counted by an experienced observer and the test was stopped after the concentration of citric acid eliciting at least 5 consecutive coughs (C5) was reached. Log transformed data are presented with error bars indicating mean +/− S.E.M. Reflex sensitivity was not significantly different between lymphocytic and non-lymphocytic patient groups (Student’s T-test).

The mean (S.E.M.) concentrations of citric acid inducing five coughs were 0.82 M (0.40) and 0.62 M (0.39) for non-lymphocytic and lymphocytic patient groups respectively, p = 0.568 (Figure 2). One patient from each of the lymphocytic and non-lymphocytic groups failed to reach a measureable [C5] and were assigned a maximal value of 4 M citric acid for graphing and analysis.

## Discussion

Respiratory tract infection and inflammation are well known to affect respiratory function, particularly in exacerbations of asthma and COPD, but there have been few studies into the action of infection on cough. We have not been able to show, based on our limited sample size, any data which might suggest an ongoing or latent respiratory infection in these patients, irrespective of the presence or absence of lymphocytic inflammation. We have also shown that BAL lymphocytosis does not appear to be related to cough reflex sensitivity in this study group.

Pathogens detected in the study subjects did not cause symptomatic infection, since this would either have explained their intractable cough or prevented them from undergoing a bronchoscopy. Although there have been many epidemiological studies investigating symptomatic respiratory infection, the incidence of asymptomatic carriage is less well known, although detection of persistent adenovirus and EBV has been documented in the lung [28,29]. Nevertheless, the pathogens detected in our study are reflected in other publications [30-33] and low rates of PCR detected coincident infection have also been reported by other authors [34]. For example, asymptomatic carriage of RSV and hMPV is rare. One study reported only one case of RSV and no cases of hMPV in 158 control patients [35], so it is unsurprising that these viruses were not detected in our subjects. In contrast, *Chlamydophila pneumoniae* frequently causes asymptomatic or low grade infection [36]. Serum microimmunofluroscence tests to *Chlamydophila sp*. report 64.3% seropositivity in healthy volunteers [37] and serological studies of *Chlamydophila pneumoniae* infection in COPD have found a significantly increased positivity (33%) when compared to healthy controls (7%) [38]. Overall, our study detected *Chlamydophila sp.* in 16.6% of subjects, 30% of controls but 10% of chronic cough patients, suggesting that prevalence might be lower in patients with chronic cough. There tended to be fewer detected pathogens in chronic cough group as a whole, compared with the healthy volunteer group, 20% vs 50% respectively but given the small sample size this was not significantly different. The protective effect of coughing, increased mucous production or a heightened immune state, might all account for this observation.

The numbers of detected pathogens were not different in lymphocytic and non-lymphocytic groups. The types of pathogens varied but the level of EBV infection in the lymphocytic population was very similar to that previously found in healthy volunteers by PCR [29]. Although high levels of EBV infection have been reported in patients with COPD [30], the numbers of patients investigated here was not sufficient to infer that our finding was more than a chance observation.

Although an association has been suggested between BAL lymphocytosis and autoimmune disease in some idiopathic chronic cough patients, to our knowledge the possibility that infection could be responsible for inflammation and cough reflex sensitivity has not previously been investigated. We could not find any association between the level of BAL lymphocytosis and the number of detected pathogens or cough reflex sensitivity. In keeping with this finding, there was no difference in the objective cough counts between lymphocytic and non-lymphocytic groups [see Additional file 2, implying that neither local infection nor the inflammatory process is obviously linked to cough severity. It is known that the long-term, predominantly monocytic, pathology of chronic infection differs markedly from that of the predominantly neutrophilic acute phase [39], suggestive of distinct infective activities and host responses. Indeed the mechanisms which promote cough in URTI, might be very different from those evident in chronic cough. For example, patients with chronic airway diseases might react differently to non-isomolar solutions than those with URTI [40] supporting the notion that distinct neuronal mechanisms might be important in chronic cough.

It appears that an inflammatory process may be present within the lymphocytic patient population, since neutrophils and eosinophils are also raised in this group. The presence of such inflammation is common in a proportion of chronic cough patients [21,41], although it is difficult to draw the conclusion from the data collected in our study that this inflammation is related to an infective or autoimmune process. Nonetheless, the association between lymphocytosis, auto-immune disease and the modulation of neuronal function is well established in conditions such as IBD, neuropathic pain syndromes and many other neurological conditions [42-45]. It must be noted though, that the symptoms in these diseases are often attributed to auto-immune mediated damage of peripheral nerves where some modalities of sensation can be exaggerated whilst others are lost. In IBD patients, and multiple rodent models of colitis, T-cell mediated, neutrophilic and eosinophilic, inflammation results in necrosis of enteric axons and significant neuroplasticity [46]. The subsequent expansion of surviving neurons, observed in these models [47], probably accounts for the increased numbers of TRPV1 expressing neurons [48]. A similar mechanism might account for increased TRPV1 expression in chronic cough [49]. Moreover, some post-herpetic neuralgia (PHN) patients report pain associated with healing onset rather than the appearance of lesions [50]. The mechanism by which the well documented nerve damage caused by autoimmune diseases might contribute to cough is not clear, although a form of infective or autoimmune ganglionitis is an intriguing possibility. Future studies should address this question.

To investigate the current airway pathogenic load we used PCR analysis of biopsy samples since serology documents past, but does not always indicate ongoing infection. An involvement of respiratory viruses or consequent autoimmune lymphocytosis in chronic cough cannot be completely ruled out. Firstly, this study was not powered to detect differences between controls and chronic cough patients for organisms with a high prevalence in the general population. Secondly, the effects of infection can outlast the pathogen [51] and auto-immunity itself may only become apparent after a substantial period of time. In addition, we cannot preclude the existence of a current but undetected/undetectable infection, in the lung or at a distal site not sampled. In the case of PHN, Zoster infection is thought to lead to T-cell infiltration to selective dorsal root ganglia. This inflammation is associated with significant loss of myelinated neurons and atrophy of the dorsal horn [52]. Whilst VZV might still be detected in lymphocytic infiltrates in a number of ganglia, the affected peripheral tissue is not thought to be a viral reservoir [53].

## Conclusions

This is the first study to consider the possible role of sub-clinical infection in chronic cough. Through parallel comparison of chronic cough patients, sampled and tested in the same way as healthy volunteers, we have not been able to show an association between the presence of infection and lymphocytosis or cough reflex sensitivity. Given the limited sample sizes employed in this study, further work is required to fully define the potential role for infection in the mechanisms underlying chronic cough and the peripheral or central, nervous sensitisation or neuroplastic change which might contribute to reflex hypersensitivity. Whilst our study demonstrates that it is unlikely that all chronic cough is caused by a single infection, larger sample sizes will be needed in future studies, to fully address the extent to which a proportion of chronic cough patients might suffer from ongoing respiratory infection, as one of many potential causes of this condition.

## Abbreviations

ANOVA: Analysis of variance; BAL: Bronchoalveolar lavage; COPD: Chronic obstructive pulmonary disease; EBV: Epstein-barr virus; HBoV: Human bocavirus; HCoV: Human coronovirus; hMPV: Human metapneumovirus; IBD: Inflammatory bowel disease; LREC: Local research ethics committee; NK1R: Neurokinin-1 receptor; PHN: Post herpetic neuralgia; PIV: Parainfluenza virus; RSV: Respiratory syncytial virus; SEM: Standard error of the mean; TGF-β: Transforming growth factor beta; TRPV1: Transient receptor potential cation channel subfamily V (vanilloid) 1; URTI: Upper respiratory tract infection; VZV: Varicella zoster virus.

## Competing interests

**Financial/Non-financial Disclosures:** The authors have no interests to declare.

## Authors’ contributions

PWW performed the data analysis and wrote and revised this manuscript. AK recruited study subjects and performed challenge tests and approved the final version of the manuscript. SD recruited study subjects and performed bronchoscopy procedures and approved the final version of the manuscript. WD performed PCR tests and approved the final version of the manuscript. JPS contributed to the original concept of the study, supervised the PCR testing, revised and approved the final version of the manuscript. PWB performed BAL cell counts and approved the final version of the manuscript. AAW contributed the original concept of the study and approved the final version of the manuscript. JAS contributed the original concept of the study, performed bronchoscopy procedures, revised and approved the final version of the manuscript. All authors read and approved the final version of the manuscript.

## Supplementary Material

Additional file 1 Additional methods describing the quantitative and reverse-transcriptase PCR analysis of endobronchial biopsies for the presence of nucleic acid sequences specific to pathogens of interest. Click here for file

Additional file 2 **Mean 24-hour objective cough rate for non-lymphocytic (BAL lymphocytes ≤ 10%) and lymphocytic (BAL lymphocytes >20%) chronic cough patients. **Data are presented as mean number of coughs per hour for each patient. There was no significant difference in the 24 hour cough rate between the lymphocytic and non-lymphocytic chronic cough patients (Student’s T-Test). Error bars indicate mean +/- S.E.M. Click here for file
