# Supplementary material for: PCR based bronchoscopic detection of common respiratory pathogens in chronic cough: a case control study
Source: Cough. 2012 Sep 14;8:5. doi: 10.1186/1745-9974-8-5 (PMC3496690; doi:10.1186/1745-9974-8-5)
Supplement: Additional file 1 — Additional methods describing the quantitative and reverse-transcriptase PCR analysis of endobronchial biopsies for the presence of nucleic acid sequences specific to pathogens of interest. [file 1745-9974-8-5-S1.pdf]

PCR based bronchoscopic detection of common respiratory pathogens in chronic cough: a case control study

Peter W. West, PhD [1,2], Peter.West@manchester.ac.uk

Angela Kelsall, PhD [1,2], Angela.Kelsall@manchester.ac.uk

Samantha Decalmer, PhD [1], samdecalmer@hotmail.com

Winifred Dove [4], W.Dove@liverpool.ac.uk

James P. Stewart, PhD[4], J.P.Stewart@liverpool.ac.uk

Paul W. Bishop, FRCPath[3], Paul.Bishop@uhsm.nhs.uk

Ashley A. Woodcock, MD [1,2], Ashley.Woodcock@manchester.ac.uk

Jaclyn A. Smith, PhD [1], Jacky.Smith@manchester.ac.uk

Additional Methods

Qualitative PCR was performed on biopsies for Respiratory Syncytial Virus (RSV), Human metapneumovirus (hMPV), Influenza A+B, Parainfuenza virus 1–4, Coronavirus (HKU-1, NL63, OC43), Rhinovirus, Adenovirus, *Chlamydia pneumoniae*, *Mycoplasma pneumonia*e, and Human Bocavirus (HBoV) using methods and primers as described previously[1]. Briefly, Human metapneumovirus (hMPV) was detected by reverse transcriptase polymerase chain reaction (RT-PCR) utilizing primers specific to matrix (M) gene. Respiratory syncytial virus (RSV) was detected by RT-PCR using primers specific to the N gene as described. Influenza A & B viruses were detected by RT-PCR using primers specific to negative sense (NS1) genes and parainfluenza (PIV) 1, 2, 3 & 4 were detected using primers specific to heamagglutinin-neuraminidase (HN) (PIV1-3) and type 4B phosphoprotein genes (PIV4). Rhinoviruses were detected by RT-PCR using primers specific to 5' UTR sequence. Human coronaviruses (HCoV - 229E, OC43, NL63 & HKU1) were detected by RT-PCR using primers specific to the viral replicase gene. Primers specific to adenovirus *hexon* gene, *Mycoplasma pneumoniae* and *Chalmydia sp.* 16S rRNA genes, were used to detect these agents. Human bocavirus (HBoV) was identified by PCR using corrected versions of specific primers for the gene encoding nonstructural protein-1 (NP-1).

Epstein-Barr virus (EBV) was detected using the *artus* EBV LC PCR kit (Qiagen, Crawley, UK). Varicella zoster virus (VZV) was detected by Taqman™ PCR using primers specific for the ORF28 gene (DNA polymerase) as follows: sense 5'-GCGCGGTAGTAACAGAGAATTTC-3'; anti-sense 5'-TGGGCACATCTTCATCTAAACATT-3'. Probe FAM- ACCATGTCATCGTTTCAA – MGB.

References:

1. Carrol ED, Mankhambo LA, Guiver M, Banda DL, Denis B, Dove W, Jeffers G, Molyneux EM, Molyneux ME, Hart CA *et al*: **PCR improves diagnostic yield from lung aspiration in Malawian children with radiologically confirmed pneumonia**. *PLoS One*, **6**(6):e21042.
