# Supplementary material for: PCR based bronchoscopic detection of common respiratory pathogens in chronic cough: a case control study
Source: Cough. 2012 Sep 14;8:5. doi: 10.1186/1745-9974-8-5 (PMC3496690; doi:10.1186/1745-9974-8-5)
Supplement: Additional file 2 — Mean 24-hour objective cough rate for non-lymphocytic (BAL lymphocytes ≤ 10%) and lymphocytic (BAL lymphocytes >20%) chronic cough patients. Data are presented as mean number of coughs per hour for each patient. There was no significant difference in the 24 hour cough rate between the lymphocytic and non-lymphocytic chronic cough patients (Student’s T-Test). Error bars indicate mean +/- S.E.M. [file 1745-9974-8-5-S2.pdf]

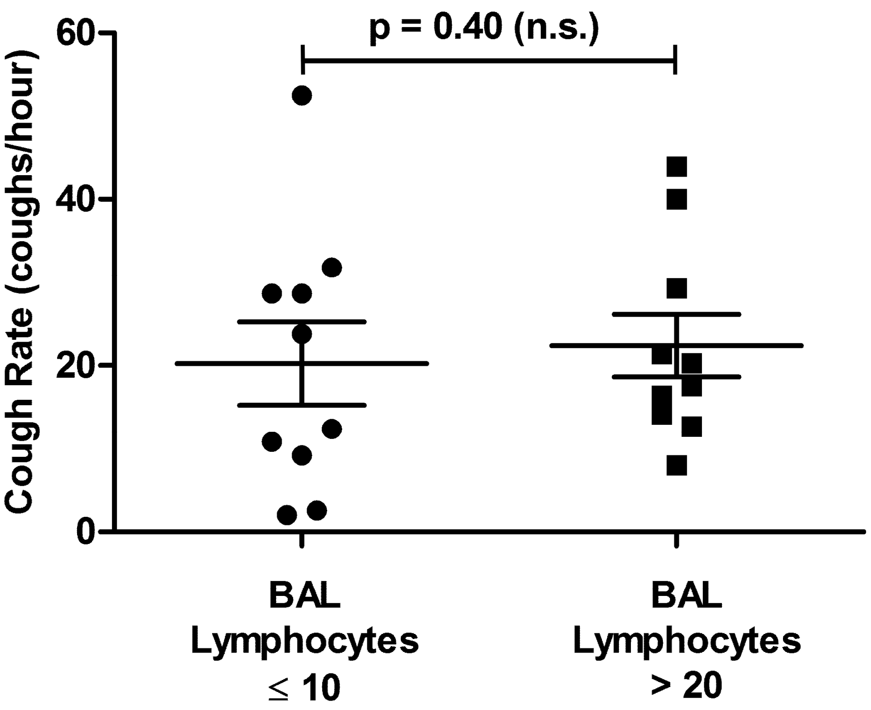

Additional File 2

Mean 24-hour objective cough rate for non-lymphocytic (BAL lymphocytes ≤ 10 %) and lymphocytic (BAL lymphocytes >20 %) chronic cough patients. Data are presented as mean number of coughs per hour for each patient. There was no significant difference in the 24 hour cough rate between the lymphocytic and non-lymphocytic chronic cough patients (Student's T-Test). Error bars indicate mean +/- S.E.M.
